# The Expression of Psoriasin (S100A7) and CD24 Is Linked and Related to the Differentiation of Mammary Epithelial Cells

**DOI:** 10.1371/journal.pone.0053119

**Published:** 2012-12-27

**Authors:** Jenny Vegfors, Stina Petersson, Anikó Kovács, Kornelia Polyak, Charlotta Enerbäck

**Affiliations:** 1 Ingrid Asp Psoriasis Research Center, Department of Clinical and Experimental Medicine, Linköping University, Linköping, Sweden; 2 Department of Pathology, Sahlgrenska University Hospital, Gothenburg, Sweden; 3 Department of Medical Oncology, Dana-Farber Cancer Institute, Harvard Medical School, Boston, Massachusetts, United States of America; University of South Alabama, United States of America

## Abstract

Psoriasin (S100A7), a member of the S100 family of calcium-binding proteins, is highly expressed in high-grade ductal carcinoma *in situ* (DCIS) and in the benign hyperproliferative skin disorder psoriasis. The gene that encodes psoriasin and many other S100 genes are located within a gene cluster on chromosome region 1q21, known as the epidermal differentiation complex. This cluster contains genes for several differentiation markers that play important roles in the terminal differentiation of the epidermis. The purpose of the present study was to evaluate the role of psoriasin in the differentiation process of mammary epithelial cells. Normal mammary epithelial cells (MCF10A) cultured in confluence and suspension, conditions known to induce psoriasin expression, demonstrated a shift towards a more differentiated phenotype indicated by an increase in the expression of the luminal differentiation markers CD24 and MUC1 and the reduced expression of the breast stem cell marker CD44. The expression of psoriasin and MUC1 was most pronounced in the CD24^+^-enriched fraction of confluent MCF10A cells. The shift towards a more differentiated phenotype was abolished upon the downregulation of psoriasin using short hairpin RNA (shRNA) and small interfering RNA (siRNA). Using specific inhibitors, we showed that psoriasin and CD24 expression was regulated by reactive oxygen species (ROS) and the nuclear factor (NF)-κB signaling pathways. While immunohistochemical analyses of DCIS showed heterogeneity, the expression of psoriasin and CD24 showed a similar staining pattern. Our findings suggest that the expression of psoriasin is linked to the luminal differentiation marker CD24 in mammary epithelial cells. Psoriasin demonstrated an essential role in the shift towards a more differentiated CD24^+^ phenotype, supporting the hypothesis that psoriasin plays a role in the differentiation of luminal mammary epithelial cells.

## Introduction

Breast tumors display a high degree of heterogeneity. Clinically, they are divided into subtypes based on protein expression patterns and clinical outcomes. Ductal carcinoma *in situ* (DCIS) is one of the earliest forms of breast cancer and is regarded as a precursor of invasive ductal carcinoma. High-grade DCIS is associated with a high risk of progression to invasive breast cancer [1].

Psoriasin (S100A7) is a member of the S100 family of calcium-binding proteins that have been shown to be involved in the regulation of many calcium-dependent cellular processes, such as proliferation, apoptosis, differentiation, invasion and metastasis [2]. Psoriasin is one of the most highly expressed genes in high-grade DCIS [3], [4]. Whereas the level is often reduced in invasive breast carcinomas, the persistent high expression of psoriasin is associated with markers of poor prognosis [5]. Psoriasin has been shown to modulate tumor growth by activating several signaling pathways. Psoriasin interacts with Jun-activating binding protein 1 (Jab1), which is involved in multiple signal transduction pathways, including the regulation of c-Jun/AP1 transcription factors [6]. Psoriasin induces epidermal growth factor signaling [7] and upregulates inflammatory pathways in breast cancer [8]. We and others have demonstrated that psoriasin expression correlates with increased survival and nuclear factor (NF)-κB signaling in mammary epithelial cells [4], [5]. Moreover, we recently showed that psoriasin is induced by reactive oxygen species (ROS) and acts through receptor for advanced glycation end products (RAGE) to induce endothelial cell proliferation and angiogenesis [9].

The epidermal differentiation complex (EDC), located on chromosome region 1q21, contains genes that play important roles in the terminal differentiation of the human epidermis, including psoriasin and other S100 genes [10], [11]. We have previously demonstrated that psoriasin is related to the terminal differentiation of keratinocytes [12]. The stimulation of differentiation *in vitro* induced the expression of psoriasin and the differentiation marker keratin I in keratinocytes [12]. Furthermore, in a spectrum of conditions of dysregulated keratinocyte differentiation, psoriasin expression correlated with the degree of keratinocyte differentiation. It has been suggested that CD44 expression characterizes mammary epithelial stem cells, while CD24 has been identified as a molecular marker of more differentiated luminal epithelial cells [13]. It has been shown that CD24 is a marker of tumor aggressiveness and that the expression of CD24 promotes breast cancer development [14], [15], [16]. Furthermore, both psoriasin and CD24 have been associated with an unfavorable prognosis for patients with breast cancer [5], [6], [16], [17], [18]. As both breast and skin epithelial cells have ectodermal origins, the induction and functional relevance of psoriasin may be similar in these two epithelial cell types.

Here, we demonstrate an association between psoriasin and CD24 in mammary epithelial cells, along with a similar staining pattern *in vivo*. These data support the hypothesis that psoriasin plays a role in the differentiation of mammary epithelial cells.

## Materials and Methods

### Ethics statement

The study was performed under conditions approved by the Ethics Committee at the University of Gothenburg, Gothenburg, Sweden. Cases were selected from the archives of the Department of Pathology, Sahlgrenska University Hospital, Gothenburg, Sweden. The study was submitted to the local Institutional Review board and complied with institutional guidelines. Immunohistochemical stains were created from paraffin blocks in a strictly anonymized fashion, therefore a written consent was not mandatory. Instead, patients gave their verbal informed consent which was documented in the referral. The consent procedure was approved by the ethics committee.

### Cell lines and culture conditions

The normal immortalized human mammary epithelial cell line, MCF10A, was obtained from the American Type Culture Collection (ATCC) and cultured as previously described [4]. The establishment of stable clones with downregulated psoriasin expression was made by transfecting MCF10A with short hairpin RNAs (shRNAs) directed against human psoriasin, as previously described [19], [20]. Transient downregulation of psoriasin expression was accomplished by transfecting MCF10A cells with small interfering RNA (siRNA) directed against psoriasin (sc-106459, Santa Cruz Biotechnology, Santa Cruz, USA), according to the manufacturer's instructions. For confluence culture, cells were maintained in the confluent condition for 5 or 10 days. Suspension culture was achieved by plating cells in poly-2-hydroxy-ethylmethacrylate (polyHEMA)-coated (10 mg/cm^2^ in 95% ethanol) Petri dishes for 3 days. Recombinant psoriasin protein (Abnova, Taipei, Taiwan) was added to a final concentration of 2.7 µg/ml and the cells were incubated for 48 hours. MCF10A cells in confluence were treated, every other day for ten days, with N-acetyl-cysteine (NAC) (10 mM), caffeic acid phenethyl ester (CAPE) (50 µM), Tyrphostin (10 µM), U73122 (10 µM), Wortmannin (50 nM) and DMSO (diluents control), all purchased from Sigma-Aldrich (St Louis, USA), or infected with dominant negative IKK-beta (dnIKKB) adenoviral vector [21].

### Magnetic activated cell sorting (MACS)

CD24^+^ cells were isolated from confluence cultures, using magnetic activated cell sorting (MACS) (Miltenyi Biotec, Auburn, USA). A total number of 2×10^7^ cells were incubated with mouse anti-human CD24 (1∶20) (BD Bioscience, San Jose, USA) for 20 min at 4°C. The cells were magnetically labeled with goat anti-mouse IgG Micro Beads and separated on an LS column and a QuadroMACS Separator by magnetic force. Sorting was performed according to the manufacturer's instructions with the following modification: PBS containing 2 mM EDTA was supplemented with 0.5% BSA during antibody and micro bead incubation.

### Flow cytometry

The expression of CD24, CD44 and MUC1 on the cell surface was measured by double staining using flow cytometry. A total number of 3×10^5^ cells were suspended in PBS and incubated with antibodies for 30 min at 4°C. The antibodies used were FITC-conjugated mouse anti-human CD24 (1∶20), PE-conjugated mouse anti-human CD44 (1∶50) and FITC-conjugated mouse anti-human CD227 (MUC1) (1∶30), all purchased from BD Bioscience. Flow cytometry was performed using the FACSAria and the results were analyzed using the FACSDiva Software v.6.1.3 (BD Bioscience).

### Western blotting

Western blotting was performed as previously described [22]. The primary antibodies that were used were the specific mouse anti-psoriasin (IMG-409A, Imgenex, San Diego, USA) [23] and rabbit anti-GAPDH (Santa Cruz Biotechnology, Santa Cruz, USA). Image analysis was performed using a Fuji film LAS-1000 camera (Las1000, Image-Gauge, Fuji, Tokyo, Japan). Protein expression was quantified using the Multi Gauge v 3.0 software.

### Tissue samples

Tissue samples from 21 DCIS tumors, selected at random, were obtained from the files of the Departments of Dermatology and Pathology, Sahlgrenska University Hospital, Gothenburg, Sweden. Blocks of formalin-fixed and paraffin-embedded tissue samples were obtained.

### Immunohistochemistry

A specific monoclonal anti-S100A7 antibody (1∶200) (IMG-409A, Imgenex) [23] and a monoclonal anti-CD24 antibody (1∶50) (MA1-19725, clone SN3) (Pierce Biotechnology, Rockford, USA) were incubated for 25 min at room temperature. Antigen-antibody complexes were detected by using an ABC detection system (Vectastain Elite, P K-6102) and diaminobenzidine (DAB) as the chromogen. All slides were evaluated by an experienced breast pathologist (AK). Tumors were considered to be positive for psoriasin and CD24 when neoplastic cells displayed distinct brown nuclear, cytoplasmic or membranous staining.

### Statistical analysis

Statistical significance was determined by the Student's t-test. Correlation analysis was performed by Spearman's rank correlation test (r). A value of p<0.05 was considered statistically significant. The results are presented as the mean ± standard deviation (SD) for at least three independent experiments.

## Results

### Induced psoriasin expression is associated with increased CD24 expression

**Figure 1:**
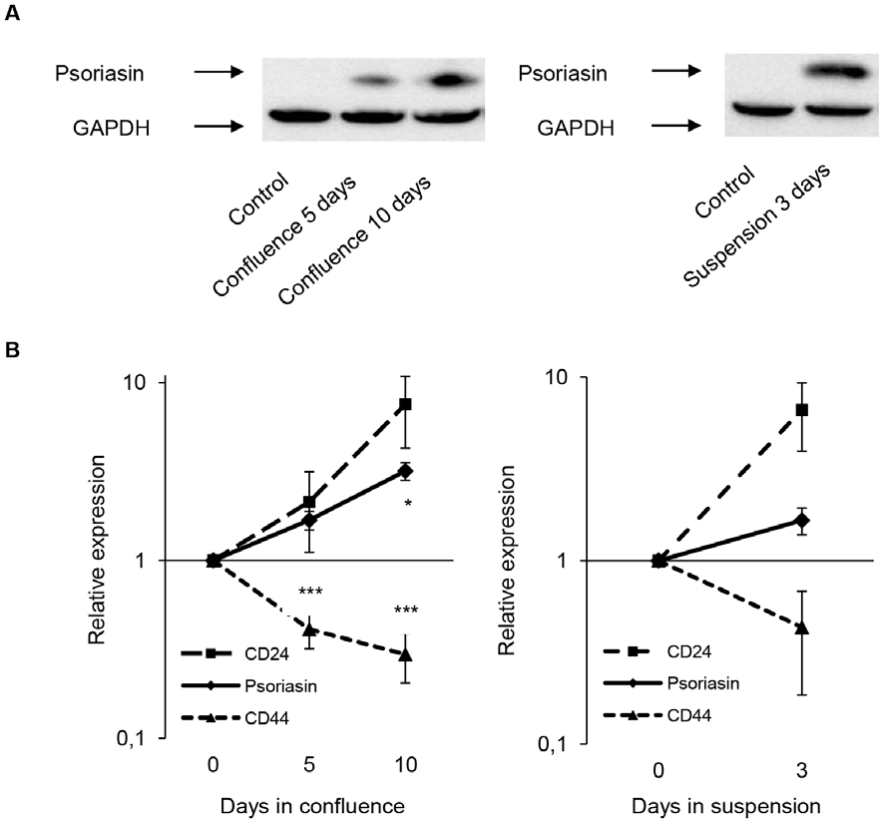
Confluence- and suspension-cultured mammary epithelial cells demonstrate increased psoriasin and CD24 expression. MCF10A cells were cultured in confluence for 5 and 10 days or in suspension for 3 days. **A** Psoriasin expression, analyzed by Western blotting, was induced in MCF10A cells cultured in confluence and suspension, compared with exponentially growing cells. Equal loading was confirmed by GAPDH. The figure illustrates a representative example (n = 3). **B** In confluence- and suspension-cultured cells, psoriasin and CD24 expression were increased, whereas CD44 expression was decreased compared with exponentially growing cells. The expression level of CD24 and CD44 was measured using flow cytometry and the expression level of psoriasin was quantified from Western blots. The data are presented as the mean ± SD of relative expression (n = 3). The p-values (*<0.05, **<0.01, ***<0.001) were calculated using the Student's t-test.

To investigate the relationship between psoriasin and the differentiation of normal mammary epithelial cells, we analyzed the expression of CD24, a marker of differentiated luminal epithelium, and CD44, a breast stem cell marker, in confluence- and suspension-cultured MFC10A cells, conditions known to induce psoriasin expression (Figure 1A). Confluence-cultured MCF10A cells showed a progressive increase in the expression of psoriasin and CD24, whereas the level of CD44 decreased (Figure 1B). Similarly, suspension-cultured cells showed an increase in the expression of psoriasin and CD24 and a decrease in CD44 (Figure 1B). The expression of psoriasin and CD24 was positively correlated in confluence- (Spearman's correlation coefficient r = 0.833, p = 0.005) and suspension-cultured cells (Spearman's correlation coefficient r = 0.841, p = 0.036). The expression of MUC1, a marker of differentiated luminal epithelia, was also increased in these culture conditions (data not shown). These results show an increase in psoriasin expression by confluence- and suspension-cultured MFC10A cells accompanied by a shift towards a more differentiated CD24^+^ phenotype. The positive correlation between the expression of psoriasin and CD24 may imply a causal relationship or be the result of differentiation.

### The expression of psoriasin and MUC1 is confined to CD24^+^ mammary epithelial cells

**Figure 2:**
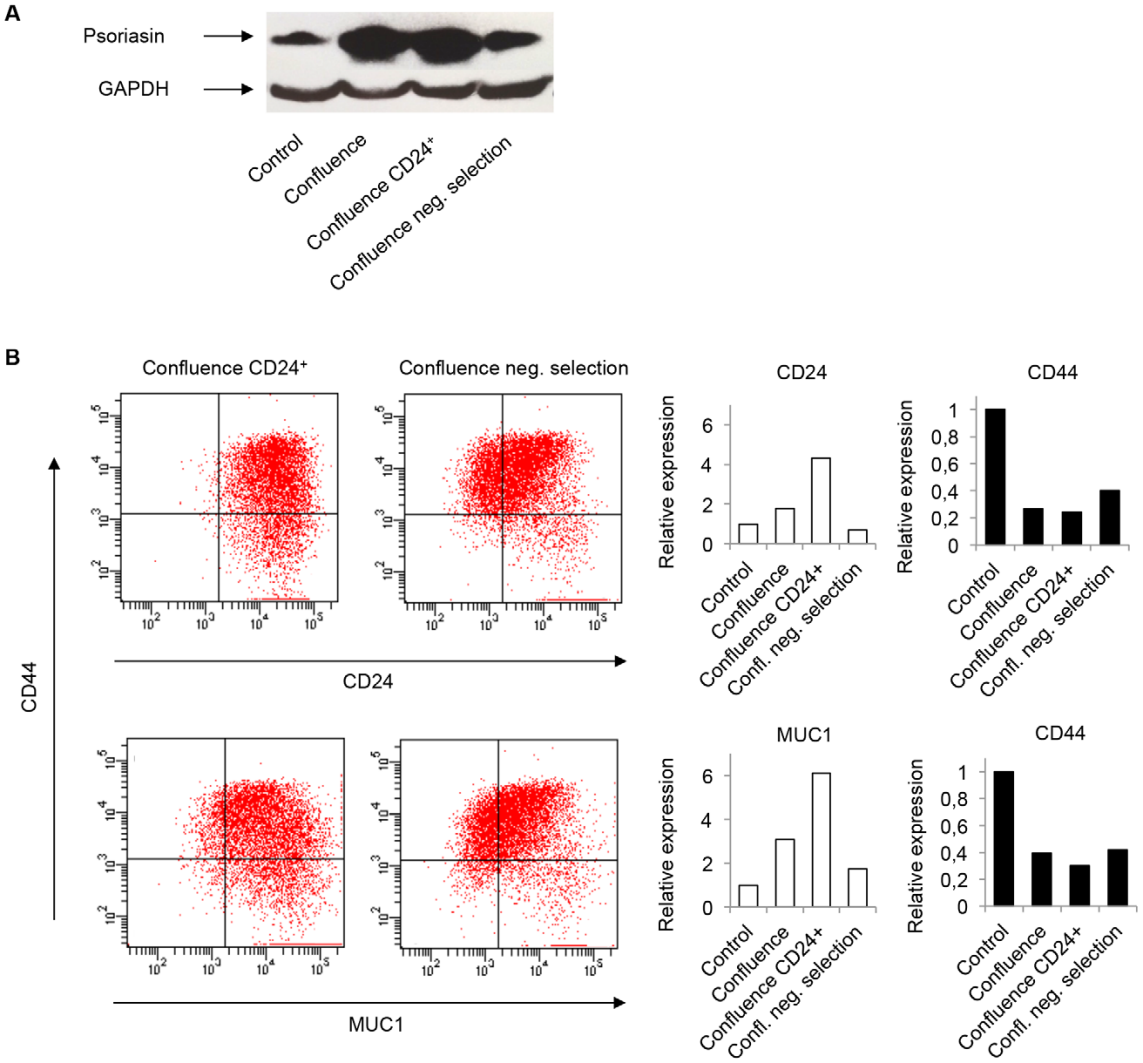
Psoriasin and MUC1 expression is elevated in CD24^+^ mammary epithelial cells. MCF10A cells were cultured in confluence for 10 days and separated for CD24^+^ cells, using magnetic activated cell sorting. **A** Psoriasin expression, analyzed by Western blotting, was confined to the CD24^+^ cell fraction, compared with negative selection and controls. Equal loading was confirmed by GAPDH. The figure illustrates a representative example (n = 3). **B** Separated CD24^+^ cells showed an increase in the expression of CD24 and a decrease in the expression of CD44, compared with negative selection. The expression of MUC1 was increased in the same level as CD24 expression. The figures illustrate representative examples (n = 4).

To investigate whether the expression of psoriasin and MUC1 is enriched in CD24^+^ cells, the CD24^+^ fraction of confluence-cultured MCF10A cells was separated, using magnetic cell sorting (MACS). We demonstrated that psoriasin expression was strikingly higher in the CD24^+^ cell fraction, compared with negative selection (Figure 2A). The expression of CD44 was reduced in the CD24^+^ cell fraction, compared with the negative selection (Figure 2B). In addition, the expression of MUC1 was elevated in the CD24^+^ fraction to levels comparable to those of CD24 expression (Figure 2B). These results suggest that psoriasin, as well as MUC1, is predominately expressed in CD24^+^ mammary epithelial cells.

### Psoriasin and CD24 expression is regulated by ROS and through the NF-κB pathway

**Figure 3:**
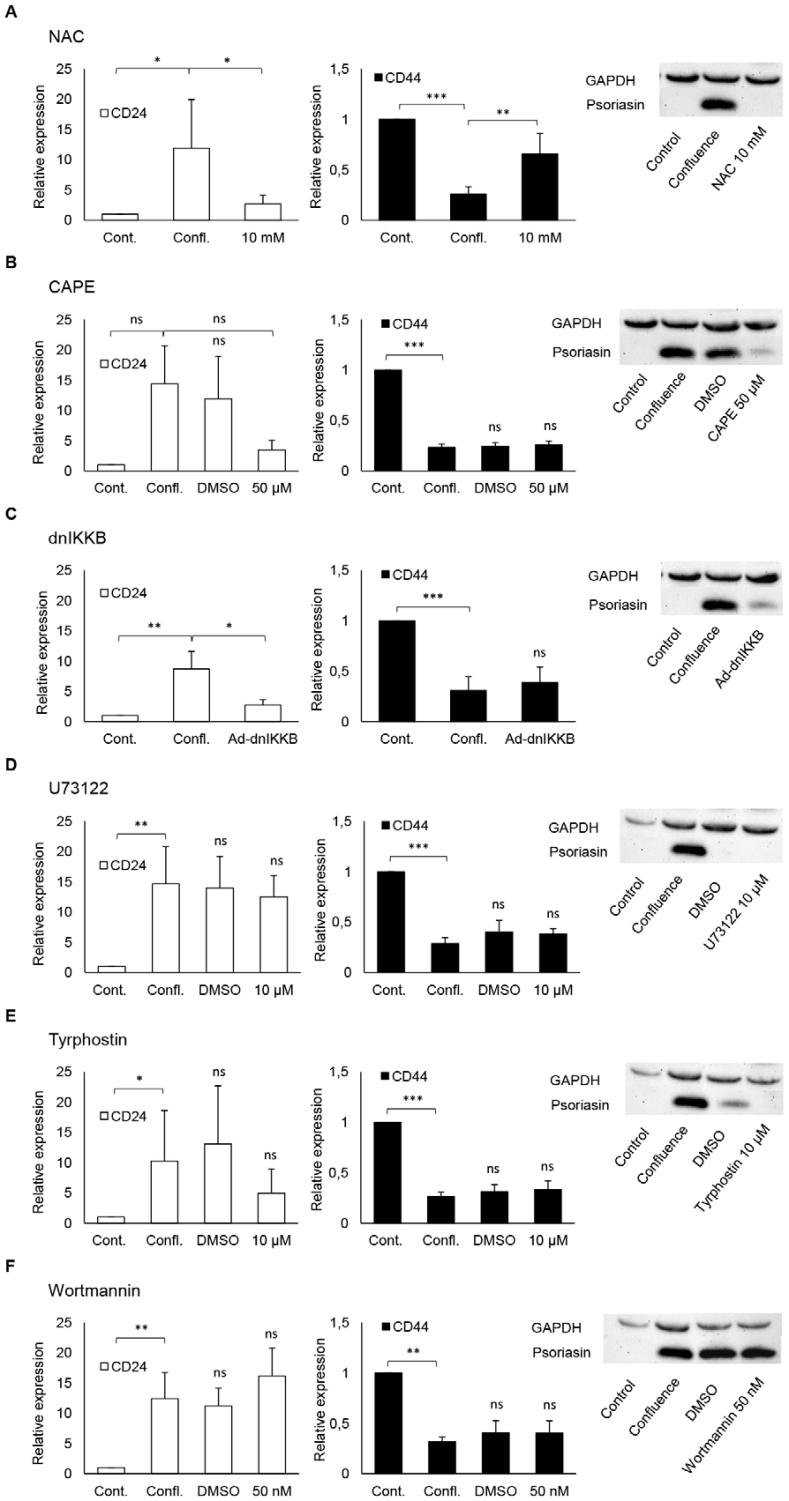
Inhibition of ROS and the NF-κB signaling pathway suppresses psoriasin and CD24 expression. MCF10A cells were cultured in confluence for 10 days. The inhibition of ROS using NAC (**A**) and NF-κB using CAPE (**B**) and dnIKKB (**C**) led to a significantly reduction in CD24 and psoriasin expression in confluence-cultured MCF10A, measured by flow cytometry and Western blot, respectively. No reduction of CD24 expression was seen by the inhibition of PLC-IP3 using U73122 (**D**). The reduced CD24 expression by Tyrphostin (**E**) did not reach statistical significance. The inhibition of PI3-K by Wortmannin (**F**) showed no decrease in psoriasin or CD24 expression. The inhibitors showed no effect on CD44 expression, except for treatment with NAC (**A**), which increased the expression of CD44. Western blot inserts illustrate representative examples (n = 4) of the psoriasin expression. Equal loading was confirmed by GAPDH. The data are presented as the mean ± SD of relative expression (n = 4). The p-values (*<0.05, **<0.01, ***<0.001) were calculated using the Student's t-test.

Since confluence culture led to a shift towards a more differentiated CD24^+^ phenotype, we set out to identify relevant signaling pathways involved in this process. We selected five signaling pathways, previously shown to be involved in the regulation of psoriasin. In confluence-cultured MCF10A cells treated with the antioxidant NAC (Figure 3A), the expression of psoriasin and CD24 was not induced, as compared to confluence control. Likewise, cells treated with inhibitors of NF-κB, CAPE (Figure 3B) and dn-IKKB (Figure 3C) did not upregulate the expression of psoriasin and CD24. Treatment with the PLC-inhibitor U73122 did not reduce the expression of CD24 (Figure 3D). Treatment with the EGFR-inhibitor Tyrphostin downregulated the expression of CD24 but this effect did not reach statistical significance (Figure 3E). The slightly reduced psoriasin expression in response to treatment with U73122 (Figure 3 D) and Tyrphostin (Figure 3E) may also depend on the high dose of DMSO used. MCF10A cells treated with Wortmannin, an inhibitor of the PI3K pathway, caused no decrease in CD24 or psoriasin expression (Figure 3F). The inhibitors had no effect on CD44 expression, except for treatment with NAC, which increased the expression of CD44. These findings suggest that the expression of both psoriasin and CD24 is regulated by ROS and the NF-κB pathway.

### Endogenous but not extracellular psoriasin causes increased CD24 expression

**Figure 4:**
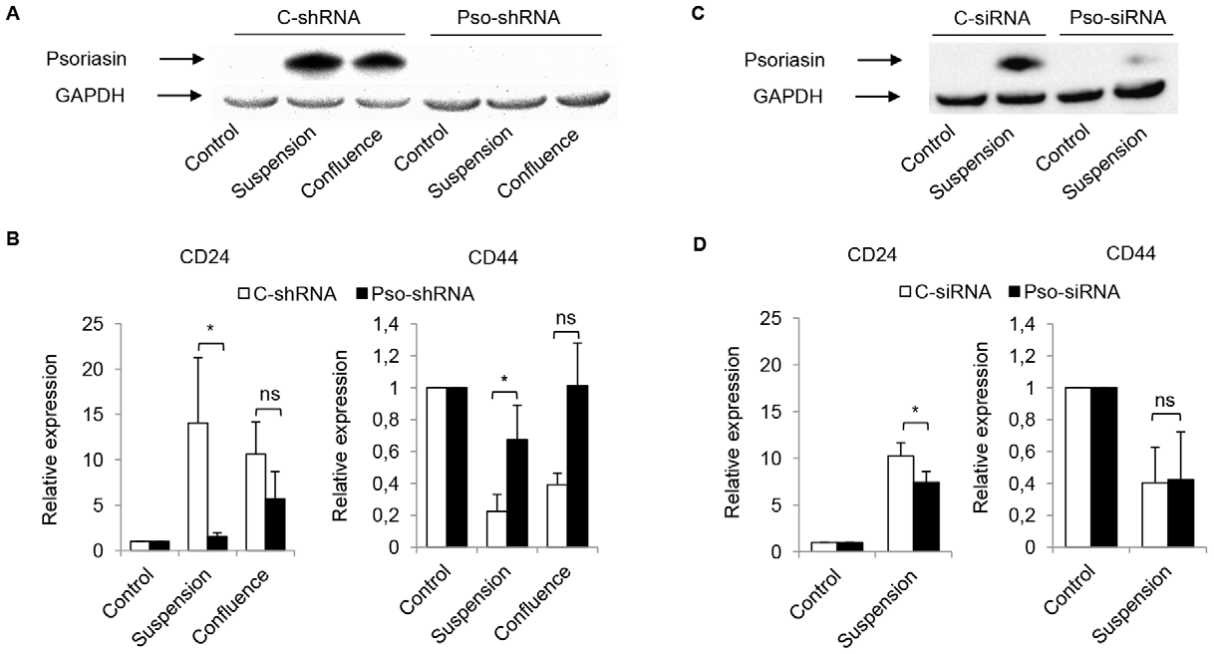
Endogenous psoriasin causes increased CD24 expression. MCF10A cells were transfected with a psoriasin-targeting shRNA (Pso-shRNA) or siRNA (Pso-siRNA), and their corresponding controls,(C-shRNA and C-siRNA). Transfected MCF10A cells were cultured in confluence for 10 days or in suspension for 3 days. The expression level of psoriasin was detected by Western blot. CD24 and CD44 expression were measured using flow cytometry. **A** No induction of psoriasin expression was observed in Pso-shRNA, compared with C-shRNA, in confluence or suspension. **B** Pso-shRNA showed a decrease in the expression of CD24 and an increase in the expression of CD44, compared with C-shRNA, during confluence and suspension. **C** The expression of psoriasin in Pso-siRNA was dramatically downregulated, compared with C-siRNA. **D** Pso-siRNA in suspension culture showed a reduced CD24 expression, compared with C-siRNA. Equal loading was confirmed by GAPDH. The figures illustrate representative examples (n = 3). The data are presented as the mean ± SD of relative expression (n = 3). The p-values (*<0.05) were calculated using the Student's t-test.

To evaluate the role of psoriasin in the regulation of CD24 and MUC1 expression, the endogenous expression of psoriasin in MCF10A cells was downregulated by shRNA and siRNA. The downregulation of psoriasin expression was confirmed in MCF10A Pso-shRNA (Figure 4A) and Pso-siRNA (Figure 4C) compared with C-shRNA and C-siRNA, in suspension culture. Next, we cultured MCF10A Pso-shRNA and Pso-siRNA in confluence or in suspension. Cells in which psoriasin upregulation was prevented displayed a reduction in the expression of CD24, along with an increase in the expression of CD44, compared with C-shRNA and C-siRNA (Figure 4B and 4D). No significant difference in MUC1 expression was observed (data not shown). We and others have previously shown that psoriasin is secreted by mammary epithelial cells [4], [24] and may act through RAGE to promote endothelial cell proliferation [9]. The treatment of MCF10A cells with purified recombinant psoriasin protein did not change the expression of CD24, CD44 and MUC1 compared with untreated MCF10A cells (data not shown). These results suggest that endogenously expressed psoriasin plays a crucial role in the shift towards a CD24^+^ phenotype.

### Psoriasin and CD24 demonstrate a similar staining pattern in DCIS breast tumors

**Figure 5:**
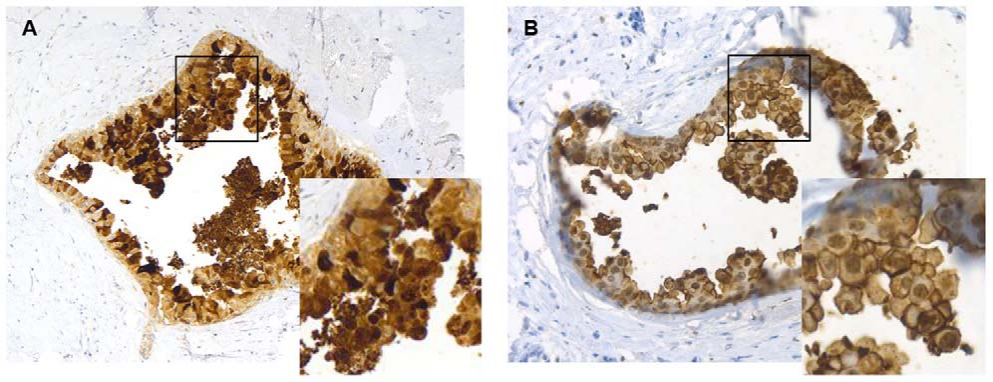
Psoriasin and CD24 demonstrate a similar staining pattern in DCIS. The expression patterns of psoriasin and CD24 in ductal carcinoma *in situ* (DCIS) were analyzed by immunohistochemisty. Psoriasin and CD24 showed an extremely intense staining and a similar staining pattern in DCIS. **A** Psoriasin staining was observed in the cytoplasm and the nucleus of the cells. **B** CD24 staining was observed in the cytoplasm, the nucleus and in the membranes of the mammary epithelial cells. The figures illustrate representative examples of psoriasin and CD24 expression in the same DCIS tumor.

We used immunohistochemistry to examine the expression pattern of psoriasin and CD24 *in vivo*. The expression of psoriasin and CD24 in a set of DCIS cases showed heterogeneity both between and within samples. Psoriasin staining was observed in the cytoplasm and the nucleus of the cells, whereas CD24 staining was observed in the cytoplasm, the nucleus and in the in the membranes of the mammary epithelial cells of the DCIS tumors. Psoriasin (57.1%; 12/21) and CD24 (47.6%; 10/21) staining was detected with a weak to strong expression in the DCIS cells. Interestingly, psoriasin and CD24 expression showed a similar staining pattern among the tumors. A concordant expression was seen in 61.9% (13/21) of cases of which ten cases showed double-positive and three cases double-negative expression. In two out of the 21 investigated cases, normal breast tissue showed a faint psoriasin staining. None of the analyzed cases showed positive staining of CD24 in normal breast tissues. As demonstrated in Figure 5, psoriasin and CD24 showed an extremely intense staining and similar staining pattern *in vivo*. These findings confirm the association between psoriasin and CD24 expression observed in vitro.

## Discussion

Psoriasin is one of the most abundantly expressed proteins in high-grade DCIS, compared with normal mammary epithelium and invasive breast carcinomas [4], [25], [26], [27]. The persistent high expression of psoriasin in invasive breast carcinoma is associated with a poor clinical outcome [5]. We previously showed that, in human immortalized mammary epithelial cells, MCF10A, psoriasin is upregulated by a loss of attachment to the extracellular matrix and prolonged confluence [4], [21], [22]. Both these conditions mimic the *in vivo* situation that is likely to occur in a high-grade comedo DCIS tumor environment, suggesting a specific role for psoriasin in the initiation of these breast tumors. The induction of psoriasin, by conditions like suspension and confluence, has also been observed for skin epithelial cells (keratinocytes), along with the increased expression of the differentiation marker keratin I [28]. In fact, well-known keratinocyte differentiation stimulators include an increase in cell density (confluence) [29] and the elevation of extracellular calcium [30]. Moreover, keratinocytes cultured in suspension have also been shown to enter a differentiation program [31]. In a previous study, we compared the expression of psoriasin in a spectrum of conditions of dysregulated keratinocyte differentiation. The expression correlated with the degree of keratinocyte differentiation with absent expression in undifferentiated basalioma and strong expression in carcinoma *in situ,* as well as in keratoacanthoma and differentiated squamous cell carcinoma [28]. These immunohistochemical findings raised the hypothesis that psoriasin is involved in the differentiation process in different types of epithelium. In humans, CD24 has been identified as a molecular marker that allows a distinction to be made between differentiated luminal epithelial and myoepithelial cells [13]. Moreover, breast cancer stem cells are characterized by the low or negative expression of CD24 but the high expression of CD44 [13].

We demonstrated that the suspension and confluent culturing conditions of mammary epithelial cells induce a shift towards the more differentiated CD24^+^ phenotype, in parallel with the endogenous induction of psoriasin. We also showed a decrease in CD44 expression, a breast stem cell marker, and an increase in MUC1 expression, a luminal differentiation marker. Interestingly, we demonstrated that the degree of shifting was dependent on the expression level of psoriasin, suggesting that the shift to a more differentiated phenotype may be dependent on the level of psoriasin expression. When analyzing the CD24^+^ cell fraction of confluence-cultured mammary epithelial cells, we showed that the level of psoriasin and MUC1 was higher in this cell fraction compared with negative selection. This supports the notion that psoriasin is expressed in more differentiated mammary epithelial cells.

We have previously shown that psoriasin expression is induced by ROS and downregulated by treatment with the antioxidants Bcl-2 and NAC [21]. We now show that psoriasin and CD24 expression are both reduced in confluence-cultured MCF10A cells treated with the antioxidant NAC, which suggests that psoriasin and CD24 expression is regulated by ROS. We and others have demonstrated that psoriasin expression in mammary epithelial cells correlates with NF-κB signaling and increased cellular survival [21], [32]. NF-κB is involved in many biological processes, including proliferation, survival and differentiation. Accordingly, we now show that treatment with NF-κB inhibitors downregulates the expression of psoriasin and CD24 in confluence-cultured MCF10A cells. Psoriasin was further shown to be induced by epidermal growth factor signaling [7] and we demonstrate a trend towards the downregulation of both psoriasin and CD24 by Tyrphostin, supporting hypothesis that EGFR signaling may be implicated in their regulation.

The role of psoriasin in the differentiation of mammary epithelial cells was further supported by showing a reduced shift towards the CD24^+^ phenotype when downregulating psoriasin using shRNA and siRNA. Our data suggest that induced endogenous psoriasin expression is related to the shift towards a CD24^+^ phenotype and that psoriasin is important in the differentiation process of mammary epithelial cells. The expression of CD24 has been shown to be significantly higher in DCIS compared with normal tissue [33], [34]. We demonstrate a similar staining pattern of psoriasis and CD24 in DCIS in vivo. When a concordant expression was present, psoriasin and CD24 often displayed extremely intense staining and similar staining pattern. In apocrine metaplasia, the differentiation and transition of a normal mammary epithelium to an apocrine sweat gland, we have observed an increase in psoriasin expression [35]. The apocrine sweat gland demonstrates an increase in the expression of CD24 and a reduction in the expression of Bcl-2 [36]. Moreover, we have previously shown that the expression of MUC1 is weak to moderate in normal epithelial cells and strong in DCIS, basically corresponding to the expression of psoriasin [37]. Psoriasin and CD24 expression in breast cancer has been associated with a poor prognosis [5], [6], [16], [17], [18], [38]. Likewise, MUC1 is frequently overexpressed and associated with a poorer prognosis in many cancers including breast cancer [39].

Previous studies support the role of CD24 in breast cancer development [14]. In humans, CD24 was identified as a ligand for P-selectin, an adhesion receptor on endothelial cells and platelets [40]. Recently, the upregulation of VEGF-A and CD24 gene expression by the tGLI1 (truncated glioma-associated oncogene homolog 1) transcription factor was shown to contribute to the aggressive behavior of breast cancer cells [41]. Interestingly, we recently demonstrated that psoriasin induces the expression of VEGF-A in mammary epithelial cells [9].

The terminal differentiation of mammary epithelia, which is known as mammary gland branching morphogenesis, is partly dependent upon interaction with the extracellular matrix (ECM) [23]. Changes in and the dysregulation of the interactions between epithelial cells and the ECM generate signals for pathologic processes, such as breast tumor formation [24]. We previously demonstrated the phospholipase C (PLC)-mediated induction of psoriasin expression by the downregulation of the intercellular adhesion molecule 1 (ICAM1), with important functions in adhesion and signal transduction, supporting the hypothesis that psoriasin plays a role in differentiation [35].

In conclusion, we show that psoriasin is linked to the putative differentiation marker CD24 in mammary epithelial cells and that psoriasin plays an essential role in the shift towards a more differentiated CD24^+^ phenotype in culture conditions mimicking the *in vivo* conditions seen in high-grade DCIS. Our results indicate that psoriasin plays a role in the differentiation of mammary epithelial cells.
